# Root cause analysis underscores the importance of understanding, addressing, and communicating cold chain equipment failures to improve equipment performance^☆^

**DOI:** 10.1016/j.vaccine.2016.09.068

**Published:** 2017-04-19

**Authors:** Pat Lennon, Brian Atuhaire, Shahrzad Yavari, Vidya Sampath, Mercy Mvundura, Nithya Ramanathan, Joanie Robertson

**Affiliations:** a PATH, 2201 Westlake Avenue, Suite 200, Seattle, WA 98121, USA; b PATH, PO Box 7404, Kampala, Uganda; c Nexleaf Analytics, 2356 Pelham Avenue, Los Angeles, CA 90064, USA; d VillageReach, 2900 Eastlake Avenue East, Suite 230, Seattle, WA 98102, USA

**Keywords:** Root cause analysis, Cold chain equipment, Uganda, Mozambique, CCE, cold chain equipment, EPI, Expanded Programme on Immunization, MOH, Ministry of Health, PQS, Performance, Quality and Safety, UNEPI, Uganda National Expanded Programme on Immunization, UNICEF, United Nations Children’s Fund, WHO, World Health Organization

## Abstract

Vaccine cold chain equipment (CCE) in developing countries is often exposed to harsh environmental conditions, such as extreme temperatures and humidity, and is subject to many additional challenges, including intermittent power supply, insufficient maintenance capacity, and a scarcity of replacement parts. Together, these challenges lead to high failure rates for refrigerators, potentially damaging vaccines and adversely affecting immunization coverage. Providing a sustainable solution for improving CCE performance requires an understanding of the root causes of failure.

Project teams conducted small-scale studies to determine the root causes of CCE failure in selected locations in Uganda and Mozambique. The evaluations covered 59 failed refrigerators and freezers in Uganda and 27 refrigerators in Mozambique. In Uganda, the vast majority of failures were due to a cooling unit fault in one widely used refrigerator model. In Mozambique, 11 of the 27 problems were attributable to solar refrigerators with batteries that were unable to hold a charge, and another eight problems were associated with a need to adjust thermostat settings.

The studies showed that tracking and evaluation of equipment performance and failure can yield important, actionable information for a range of stakeholders, including local CCE technicians, the ministry of health, equipment manufacturers, and international partners such as the United Nations Children’s Fund, World Health Organization, and Gavi, the Vaccine Alliance. Collaborative efforts to systematically collect and communicate data on CCE performance and causes of failure will help to improve the efficiency and reach of immunization programs in low- and middle-income countries.

## Highlights

• Eighty-six refrigerators were evaluated for non-performance in Mozambique and Uganda.
• Communicating equipment failures resulted in actions that addressed failures.
• Systematic monitoring, failure analysis, and reporting strengthen the cold chain.

## 1 Introduction

Many vaccines are temperature sensitive and need to be kept in a cold chain, between 2 °C and 8 °C, to remain potent [1]. A number of challenges, however, can prevent cold chain equipment (CCE) from operating properly to maintain vaccines within the needed temperature range. These challenges are especially pronounced in low-resource settings and include intermittent and fluctuating electric power supplies, extreme ambient temperatures, high humidity, dust, substandard installation, inconsistent preventive maintenance, and inadequate supplies of parts for repairs [2].

Together, these challenges can lead to high failure rates for refrigerators in low-resource settings, which may reduce vaccine potency and decrease immunization effectiveness [3]. Indeed, previous studies have documented poor performance of CCE in many countries, with negative consequences for immunization programs [4], [5], [6], [7], [8], [9], [10], [11], [12]. According to a World Health Organization (WHO)-United Nations Children’s Fund (UNICEF) joint statement, 14% of CCE is nonfunctional and an additional 41% are poor performing based on data from 55 low- and middle-income countries [13]. These challenges also have financial implications because of the resulting waste of resources [14].

Temperature excursions can be an early indicator that CCE is malfunctioning. In some settings, immunization programs have successfully used continuous temperature monitoring to notify cold chain personnel and health workers when equipment begins to operate outside specifications and combined this with improved maintenance practices to improve the performance of CCE (PATH, unpublished report, 2014). However, providing a sustainable solution to continued temperature excursions for specific CCE requires an understanding of the root causes of equipment failure.

WHO Performance, Quality and Safety (PQS) department sets specifications for CCE and uses independent validation to confirm that the equipment meets PQS specifications before procurement. UNICEF Supply Division procures equipment that meets WHO PQS standards on behalf of many countries and donors. The UNICEF Supply Division also works with manufacturers to resolve specific equipment problems when reported by countries. However, information about the field performance of equipment is not systematically collected, analyzed, or publicly reported, though this information would be valuable to manufacturers, donors, and countries alike.

This paper describes evaluations of the root causes of CCE failures in Uganda and Mozambique. In Uganda, the Ministry of Health (MOH) and PATH collaborated to assess 59 failed refrigerators or freezers used in the cold chain. Using remote temperature monitoring, in Mozambique, the MOH, Nexleaf Analytics, and VillageReach investigated 83 refrigerators in use across health facilities in one province, and followed up in person or remotely to address 27 refrigerator failures that were detected.

The findings underscore the value of collecting and analyzing data on the root causes of CCE failure and communicating the findings to relevant audiences—such as Expanded Programme on Immunization (EPI) staff, equipment manufacturers, procurement agencies, and donors—so that corrective actions can be undertaken. The resulting information may also be useful for informing global policy, equipment design, procurement, and country-level cold chain improvements. If relatively few failure mechanisms are found to cause most equipment failures, as hypothesized based on the Pareto principle, the performance of CCE could be significantly enhanced with a relatively small number of improvements.

## 2 Methods: root cause analysis of CCE failures

Root cause analysis is a set of approaches that can be applied to determine the underlying cause(s) of a problem or failure in equipment design and production quality as well as supporting management processes [15], [16]. Each of the root cause analysis approaches is valuable to inform a broader system-level understanding of recurring challenges. Root cause analysis has previously been applied to many manufacturing issues, including problems with refrigerators [17], [18].

In Uganda, the focus of the study was on the design and production quality of failed CCE. PATH assembled a team consisting of a CCE maintenance expert, a staff member from the Uganda National Expanded Programme on Immunization (UNEPI) at the MOH, and a PATH project manager to diagnose equipment failures over a three-week period starting in June 2015. The team reviewed maintenance records and consulted MOH staff to locate equipment that was nonfunctional or operating outside WHO temperature specifications.

The Uganda study was designed to target equipment less than eight years old. However, because available national inventory data on installed equipment were incomplete, and only a few devices could be identified through those records, the project team restricted the sample of failed refrigerators to those with verification of procurement after 2006. During the past eight years, UNEPI has received approximately 600 Dometic model RCW 42 EG absorption refrigerators and 240 Dulas Solar VC-65F refrigerators. Unfortunately, the exact installation dates could not be determined due to incomplete equipment recordkeeping.

The study team visited the National Medical Stores, 15 health centers, and four district vaccine stores with failed equipment, and the CCE maintenance expert assessed 59 failed refrigerators and freezers. The data collection tool for assessment included the manufacturer name, model, approximate date of installation, and suspected root cause(s) of failure based on the diagnosis and investigation done by the CCE maintenance expert.

In Mozambique, the focus of the study was on the processes in place to support functioning equipment. Nexleaf Analytics worked with VillageReach and the Mozambique MOH, along with a CCE maintenance expert, to determine the root cause(s) of refrigerator failure and then fix all failures, where feasible, in Gaza Province. The objective was to definitively identify the primary cause of failure by fixing the problem and following up to ensure the failure was indeed addressed.

A remote temperature monitoring system was installed inside every refrigerator in Gaza Province as part of a pilot implementation with the MOH started in August 2014, and continuous temperature and power availability data were automatically transmitted from each refrigerator to a centralized dashboard. The project team, which included a maintenance technician from the MOH who was stationed at the province, reviewed three months of data to evaluate the performance of all 83 refrigerators in the province. CCE with repeated temperature excursions outside of the WHO-recommended range (2–8 °C) were identified for follow-up. The average year of installation for all refrigerators in this assessment was 2010. Among the 17 RCW 50 DC model refrigerators, installation year ranged from 2008 to 2014. Other models included in the assessment were the RCW 50 AC, RCW 50 EK, RCW 50 EG, and RCW 42 EK.

The team identified 27 refrigerators in Mozambique that required maintenance attention. The CCE expert and technician subsequently visited 12 facilities to diagnose and address failures. Failures at an additional 15 facilities were remotely diagnosed by analyzing unique temperature data patterns on the dashboard associated with either flat batteries (batteries unable to hold a charge) or improperly adjusted thermostats. The technicians addressed instances of improper thermostat adjustment by phone with facility staff, then verified the repairs were made by monitoring refrigerator performance for 72 h on the dashboard. In some cases, refrigerators could not be repaired due to lack of necessary parts, such as new batteries or compressors. In those cases, field visits were conducted over a three-week period starting in September 2015, and a survey tool was used to collect CCE manufacturer, model, root cause, and follow-up actions taken to resolve the problem.

## 3 Results of analyses in Uganda and Mozambique

The two root cause analyses found three main causes of equipment failure:

• Inherent shortcomings in equipment design or manufacturing.
• Failure to perform preventive maintenance.
• Failure to conduct corrective action once failures were identified.

Specific results in each country are outlined below.

### 3.1 Uganda

The most common cause of failure among the sampled refrigerators in Uganda was a faulty cooling unit, leading to failure of 44 of 59 units. All of the faulty cooling units were associated with a single model, suggesting a manufacturing defect. Refrigerant leaks were the second most common cause of failure. Table 1 summarizes the causes of failure as determined by root cause analysis.

The failure modes listed in Table 1 are described below:

• Cooling unit fault: A failure in the sealed cooling unit of an absorption refrigerator. The exact problem is difficult to diagnose, and repair requires replacing the entire cooling unit.
• Refrigerant leak: A leak in the evaporator or elsewhere in the cabinet.
• Control unit fault: A failure of the electronic control unit that turns the compressor on and off using a signal from the thermostat.
• Thermostat fault: A failure of the thermostat that opens or closes the compressor on/off circuit depending on the set point and internal temperature.
• Stolen solar panel: Solar panels removed and thus no longer available to provide power to the refrigerator.
• Blocked gas tube: Blockage of the tube that goes from the liquid petroleum gas canister to the refrigerator.

### 3.2 Mozambique

In Mozambique, the most common causes of failure were flat batteries in solar refrigerators and improper adjustment of thermostats. Of the 27 pieces of CCE investigated, 11 solar refrigerators required new batteries, and eight failures were addressed simply by adjusting the thermostat (Table 2).

The failure modes listed in Table 2 are described below:

• Flat battery: The battery of the solar refrigerator is unable to hold its charge. The CCE compressor operates only during the day when the solar panel is providing a charge and stops operating when the sun sets.
• Improper thermostat adjustment: The refrigerator runs too cold or too warm, and a thermostat adjustment brings the temperature back into the WHO-recommended range of 2–8 °C.
• Failed compressor: The compressor is defective and no longer working.
• Power outage: No electricity is being supplied to the refrigerator.
• Poor wiring connection: A wire that is not connected properly (for example, loose), which leads to intermittent connection and poor performance.
• Poor installation of the solar panel: Solar panels not installed to recommended best practice, including poor wiring, loose connections, and unprotected wires hanging under the panels, which could lead to unintentional disconnection.

After the thermostats were properly adjusted, there was a 30% increase in the number of refrigerators at the eight facilities that remained between 2 °C and 8 °C. Temperature excursions to greater than 8 °C declined by 78%, and excursions to less than 2 °C fell by 60%.

**Fig. 1:**
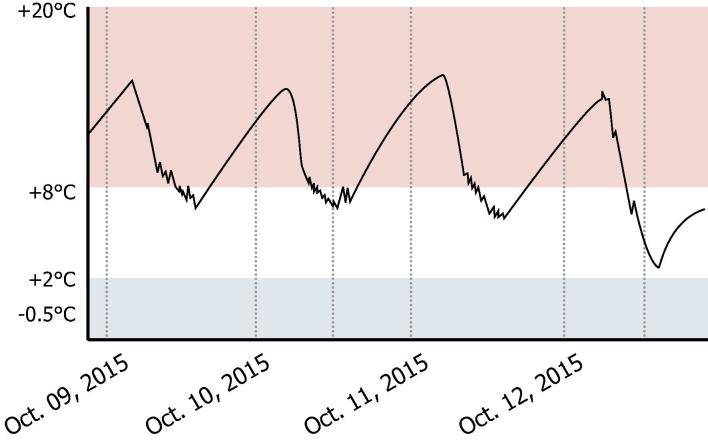
Flat battery performance of a RCW 50 DC refrigerator.

Refrigerators with flat batteries showed a distinctive pattern of temperature excursions. The temperature dropped during the day when the compressor was powered by the sun and increased at night when the flat batteries could not power the compressor to cool the refrigerator (see Fig. 1). In four facilities, the CCE expert found the solar panels to be dirty and poorly maintained. Dirty panels can lead to decreased battery charge and poor performance.

## 4 Discussion

Findings from evaluation of CCE performance and causes of equipment failure can be used at multiple levels to improve the efficiency and effectiveness of immunization programs.

At the local level, for example, CCE technicians can use findings to better manage maintenance and replacement of refrigerator parts. Technicians can prioritize visits to target the worst-performing fridges in the area, enabling efficient use of limited human resources and transportation funding. Findings can also help technicians determine which spare parts and tools they need to keep in supply, and in what quantities. Findings may also document a need to address specific installation issues, such as problems with theft of solar panels.

Access to remote temperature monitoring data may enable technicians to remotely diagnose some refrigerator failures and then communicate with facility staff to explain how to fix some easily resolved problems, such as improper thermostat settings. This could potentially help save time and money.

At the national level, evaluation data can be used for improving CCE budget planning, procurement, and placement, as well as for managing maintenance and replacement plans. For example, data on how different equipment models perform can enable the MOH to calculate the average annual cost per refrigerator model and make evidence-based replacement decisions. The failure and repair information can also be used to facilitate informed decision-making around procurement of spare parts.

Other examples of national-level uses of performance data include:

• Sending a description of the failure and the serial number of the failed equipment to the manufacturer in a timely manner, especially when the equipment is under warranty. This will be particularly useful in cases in which a manufacturing defect is suspected.
• Setting national policy and guidance related to certain issues, such as the potential need to procure refrigerators with voltage regulators to prevent adverse consequences of unreliable electrical power supplies.

Performance data can serve many purposes at the global level. For example, manufacturers can use post-sales surveillance data that include root cause analysis of faulty equipment to improve product design and quality. If problems are detected and corrected quickly, manufacturers can limit issues to a small number of production runs. Manufacturers may also consider performing small-scale field validation studies prior to large-scale order fulfilment, when new equipment is introduced or old equipment undergoes significant manufacturing or design changes, to uncover any unexpected problems.

Country-based root cause analysis informed by CCE temperature performance data may be useful to international organizations such as UNICEF, Gavi,^1^ the Vaccine Alliance, and WHO. Gavi and UNICEF, for instance, may use the data to inform procurement of equipment and spare parts. WHO may use the data to update specifications for CCE. Other potential uses of the analysis include information-sharing about CCE performance with manufacturers and other buyers (without sacrificing procurement principles and required confidentiality) and supporting further studies to inform spare parts inventory requirements. It should also be noted that the CCE performance data are the property of the local government, and use of these data require appropriate permission.

The consequences of not monitoring cold chain performance, not conducting root cause analysis, and thus not reporting equipment failures can result in a cold chain that could compromise vaccine potency, lead to increased wastage if vaccines are correctly identified by their vaccine vial monitor as being exposed to excessive heat, and potentially result in missed or under immunized children.

In the case of our pilot studies in Uganda and Mozambique, the communication of findings to stakeholders has already led to action by manufacturers and country EPI teams. Manufacturers have expressed interest in investigating the failed equipment in Uganda, and one manufacturer has offered to make repairs even though warranties have expired. Furthermore, UNEPI has updated its policy to provide voltage regulators for all mains-powered equipment to protect refrigerator electronic control units from electrical surges. One solar battery and five failing refrigerators have been replaced so far in Gaza Province in Mozambique. The MOH technician continues to replace the failing refrigerators identified. The findings of these assessments need to be considered in light of a number of study limitations. For example, these studies used purposeful sampling to identify equipment performing outside specifications, so findings concerning equipment quality and cold chain performance cannot be more broadly generalized to Uganda and Mozambique. Also, sample sizes were small. In addition, because of differences among countries in immunization programs and systems for managing CCE, findings from these two countries may not be representative of CCE failures in low-resource settings in other countries.

Moving forward, these types of studies may be improved by:

• Creating and using a standard failure definition list.
• Following a rigorous method for determining the root cause of failure (e.g., the 5 Why’s) [16], [19].^2^
• Collecting and reporting serial numbers for each piece of failed equipment.
• Broadening the root cause approach to include preventive maintenance, corrective action, and other systemic CCE challenges.

Our studies in Uganda and Mozambique have shown that tracking and evaluation of equipment performance and failure can yield important, actionable information for a range of stakeholders, including local CCE technicians, ministries of health, manufacturers, and international organizations such as UNICEF, Gavi, and WHO. Especially because of increased costs and the growing complexity of immunization supply chains, it is critical for country-level and global stakeholders to invest time and resources in CCE management data systems and prompt communication of data. Collaboration by ministries of health, manufacturers, nongovernmental organizations, funders, and international agencies to systematically and routinely collect data on CCE performance and causes of failure should be part of strengthening equipment maintenance programs, and will help to further improve the efficiency and reach of immunization programs in low- and middle-income countries. Future studies are needed to clarify the capacities and limitations of local governments to respond to underperforming equipment. Data from these studies should be used to inform the design of sustainable monitoring and repair systems.

## Funding source

This work was funded in whole or part by a grant from the Bill & Melinda Gates Foundation. The views expressed herein are solely those of the authors and do not necessarily reflect the views of the Foundation. Google.org (a Foundation) funded Nexleaf’s effort in conducting the field study reported in this paper.

## Conflict of interest

Nexleaf Analytics is a provider of remote temperature monitoring equipment.

## Figures and Tables

**Table 1:** Causes of failure of 59 refrigerators assessed in Uganda.

Cause of failure	Manufacturer, model (count), and device type*					
	Dometic RCW 42 EG^a^ (n = 44)	Dulas Solar VC-65 (n = 11)	Vestfrost MF 314^c^ (n = 1)	Sibir V 170 GE^b^ (n = 2)	Vestfrost MK 304^d^ (n = 1)	Total (n = 59)
	Absorption	Solar compression	Freezer, AC** compression	Absorption	AC** compression	
Cooling unit fault	44					44
Refrigerant leak		5			1	6
Control unit fault		2	1			3
Thermostat fault		2				2
Stolen solar panel		2				2
Blocked gas tube				2		2

* a, refrigerator; b, refrigerator and freezer; c, freezer; d, ice-lined refrigerator.

** AC = alternating current.

**Table 2:** Causes of failure of 27 refrigerators assessed in Mozambique.

Cause of failure	Manufacturer, model (count), and device type*					
	Dometic RCW 50 DC (n = 17)	Dometic RCW 50 AC (n = 4)	Dometic RCW 50 EK (n = 3)	Dometic RCW 50 EG (n = 2)	Dometic RCW 42 EK (n = 1)	Total (n = 27)
	Compressor	Compressor	Absorption	Absorption	Absorption	
Flat battery	11					11
Improper thermostat adjustment	3	3	1		1	8
Failed compressor	1					1
Power outage		1	2	2		5
Poor wiring connection	1					1
Poor installation of the solar panel	1					1

* All assessed devices were refrigerators.

## Footnotes

1 In 2016, Gavi implemented the Cold Chain Equipment Optimisation platform, which will help countries modernize cold chains with high-performing equipment.

2 One way to find the root cause of failure is to ask why a failure occurred five times (each “why” hones in on the cause).

## References

[1] World Health Organization (WHO). Department of immunization, vaccines and biologicals. Temperature sensitivity of vaccines. Geneva: WHO; 2006.

[2] WHO. Immunization supply chain and logistics: a neglected but essential system for national immunization programs. Call to action. Geneva: WHO; 2014.

[3] WHO and United Nations Children’s Fund (2014). EVM global data analysis 2010–2013.

[4] Mallik S., Mandal P.K., Chatterjee C., Ghosh P., Manna N., Chakrabarty D. (2011). Assessing cold chain status in a metro city of India: an intervention study. Afr Health Sci.

[5] Ateudjieu J., Kenfack B., Nkontchou B.W., Demanou M. (2013). Program on immunization and cold chain monitoring: the status in eight health districts in Cameroon. BMC Res Notes.

[6] Diamenu S.K., Bosnu G., Abotsi F., Achiano A.K., Sarpong F., Dadzie F. (2015). Why conduct effective vaccine management (EVM) assessment?. Int J Vaccine Immunizat.

[7] Rogie B., Berhane Y., Bisrat F. (2013). Assessment of cold chain status for immunization in central Ethiopia. Ethiop Med J.

[8] Matthias D.M., Robertson J., Garrison M.M., Newland S., Nelson C. (2007). Freezing temperatures in the vaccine cold chain: a systematic literature review. Vaccine.

[9] Murhekar M.V., Dutta S., Kapoor A.N., Bitragunta S., Dodum R., Ghosh P. (2013). Frequent exposure to suboptimal temperatures in vaccine cold-chain system in India: results of temperature monitoring in 10 states. Bull World Health Organ.

[10] Nelson C., Froes P., Van Dyck A.M., Chavarria J., Boda E., Coca A. (2007). Monitoring temperatures in the vaccine cold chain in Bolivia. Vaccine.

[11] Adu F.D., Adedeji A.A., Esan J.S., Odusanya O.G. (1996). Live viral vaccine potency: an index for assessing the cold chain system. Public Health.

[12] Techathawat S., Varinsathien P., Rasdjarmrearnsook A., Tharmaphornpilas P. (2007). Exposure to heat and freezing in the vaccine cold chain in Thailand. Vaccine.

[13] WHO, United Nations Children’s Fund. Achieving immunization targets with the comprehensive effective vaccine management (cEVM) framework. Geneva: WHO; 2016.

[14] Zaffran M., Vandelaer J., Kristensen D., Melgaard B., Yadav P., Antiwi-Agyei K.O. (2013). The imperative for stronger immunization supply and logistics systems. Vaccine.

[15] Tomic B., Spasojevic Brkic V. (2011). Effective root cause analysis and corrective action process. J Eng Manage Competit.

[16] Rooney J.J., Vanden Heuvel L.N. (2004). Root cause analysis for beginners. Quality Progress.

[17] Kalantri R., Chandrawat S. (2013). Root cause assessment for a manufacturing industry: a case study. J Eng Sci Technol Rev.

[18] Suk Han H., Bong Jeong W., Seong Kim M., Hoon Kim T. (2009). Analysis of the root causes of refrigerant-induced noise in refrigerators. J Mech Sci Technol.

[19] Williams P.M. (2001). Techniques for root cause analysis. Proc (Bayl Univ Med Cent).

